# Supplementary material for: Challenges and proposed solutions in making clinical research on COVID-19 ethical: a status quo analysis across German research ethics committees
Source: BMC Med Ethics. 2021 Jul 19;22:96. doi: 10.1186/s12910-021-00666-8 (PMC8287116; doi:10.1186/s12910-021-00666-8)
Supplement: Supplementary file 1 — Additional file 1. Questionnaire. Title of data: Questionnaire for the assessment of COVID-19 studies by research ethics committees. Description of data: The English translation of the original survey questionnaire for the RECs. [file 12910_2021_666_MOESM1_ESM.docx]

**Questionnaire for the assessment of COVID-19 studies by research ethics committees**

1. How many interventional COVID-19 studies have you reviewed in the areas of AMG, MPG and professional law as of 21 April 2020?
   1. AMG*: ___
   2. MPG**: ___
   3. Berufsrecht (German professional code for physicians): ___
2. How many non-interventional COVID-19 studies have been reviewed as of 21 April 2020?
   1. ___

**MAIN QUESTION:** Please answer this question in as much detail as possible. The more feedback we obtain on this question, the more meaningful our work on a FAQ list will be.

As an alternative to written feedback, we would be happy to arrange a telephone appointment with you in which you could provide us with answers to the questions of this survey orally. We would record this telephone conversation and then evaluate it in the same way as the written feedback. To arrange a telephone appointment, please contact us at [anna.sierawska@charite.de](mailto:anna.sierawska@charite.de).

1. Please describe special **challenges** that arose in the evaluation of COVID studies by your ethics committee and how you dealt with them (**solutions**):
   1. Statistics/Study quality ___
   2. Informed Consent: ___
   3. Risk-Benefit-Assessment: ___
   4. Other(s), please specify: ___
2. Do you have any **further comments** on how the work of the ethics committees can be supported in such a way that COVID-19 ethics proposals can be evaluated quickly but still with high quality to protect study participants and ensure meaningful study results?
   1. ___

Abbreviations:

*AMG: “Arzneimittelgesetz”, German drug law

**MPG: “Medizinproduktgesetz”, German medical device law
